# An analysis of the diagnoses and costs of pediatric emergency care visits: a single center study

**DOI:** 10.1186/s12913-024-10746-1

**Published:** 2024-02-27

**Authors:** Annika Kauppala, Paula Heikkilä, Sauli Palmu

**Affiliations:** 1 Faculty of Medicine and Health Technology, Tampere University, Arvo Ylpön katu 34, 33520 Tampere, Finland; 2 Tampere Center for Child, Adolescent and Maternal Health Research, Faculty of Medicine and Health Technology, Tampere University, Arvo Ylpön katu 34, 33520 Tampere, Finland; 3 Tampere University Hospital, Wellbeing Services County of Pirkanmaa, Elämänaukio 2, 33520 Tampere, Finland

**Keywords:** ED utilization, Resource use, Costs, Children

## Abstract

**Background:**

Children’s emergency care visits are common, although the costs and reasons for visits vary. This register-based study examines the costs of pediatric emergency care and the diagnoses related to visits made to the Pediatric Emergency Unit at Tampere University Hospital (Tays), Tampere, Finland.

**Methods:**

This retrospective study described pediatric emergency care visits made between September 2018 and December 2019 to a single center in Tampere, Finland. The data were gathered from medical files and from cost-per-patient software and analyzed in groups by age, season, level of treatment in the ED (primary or secondary), and hospitalization, as well as by diagnosis groups.

**Results:**

During the study period, 11,454 visits were made. The total costs were over €3,380,000 ($2,837,758), with a median cost per visit was €260 ($217.90). Higher costs were associated with hospitalization and treatment in secondary care. The most common diagnoses were respiratory tract infections, counseling, other infections, GI symptoms, and other reasons.

**Conclusion:**

Seriously ill children incur the highest costs per visit in pediatric emergency care. Respiratory tract infections are common reasons for emergency care visits, and the reasons why children come to emergency care in Finland are similar to those in other countries.

**Supplementary Information:**

The online version contains supplementary material available at 10.1186/s12913-024-10746-1.

## Introduction

There was an increase in pediatric emergency care visits from 1997 to 2013 in both the general and pediatric healthcare fields [1–4]. Globally, it has been estimated that pediatric emergency care comprises around 20–25% of all emergency care; [1, 2, 5] however, the cost of pediatric emergency care has been estimated to only account for around 10% of the total costs of emergency care [2]. 

Few studies have examined the costs of pediatric emergency care visits separated by age group, and their results are not fully comparable [6–10]. In these studies, the mean payment for an emergency room visit not requiring hospitalization was estimated to be $186–$554, depending on the patient’s insurance status [6, 8]. The costs vary greatly among diagnostic categories, as reflected by an estimate of the mean payment for pediatric trauma patients as high as $12,370 [7]. 

The reasons why children are admitted to emergency care units are very similar around the world. Upper respiratory tract infections and other concerns within the respiratory system, [2, 11, 12] as well as injuries or trauma [1, 13, 14] have been identified as the most common reasons for pediatric emergency care in multiple descriptive studies. Other common reasons include gastrointestinal tract problems and general symptoms, such as fever [1, 2, 11–14]. Fever on its own was discovered to be behind 24% of pediatric emergency care visits in Switzerland during the winter months [15].

In Finland, ED visits are free of charge for children < 18 years of age. The hospital gets reimbursed for the visit with a fixed rate based on the nature of the visit (i.e. primary care visit or referral). If a patient is admitted to inpatient treatment, the reimbursement of the ED visit is fixed.

In this retrospective descriptive register-based study, we assessed the costs of pediatric emergency care (individual patients) and examined the diagnoses related to visits made to the Pediatric Emergency Unit at Tampere University Hospital (Tays), Tampere, Finland. We focused on the diagnoses given and diagnostic measures taken and compared differences in these between different patient groups.

## Materials and methods

The Pediatric Emergency Unit of Tays Central Hospital provides emergency care for all children. Since September 3, 2018, it has served as a primary pediatric emergency care center; before that, it operated mostly as a secondary referral center. During the night, it is the primary emergency unit for all children living in the surrounding area, and during working hours, it operates as a secondary referral center for local primary healthcare clinics. Both general physicians and pediatricians are present to provide medical help for children with emergent conditions. Children suffering from severe trauma are treated in the adult unit located on the same hospital campus. Tays Central Hospital is the largest campus of Tays University Hospital, with 87,000 annual emergency department visits. There are approximately 112,000 children < 19 years old in the Pirkanmaa Hospital District, and the Pediatric Emergency Unit of Tays Central Hospital is responsible for the pediatric emergency care of these children.

**Fig. 1:**
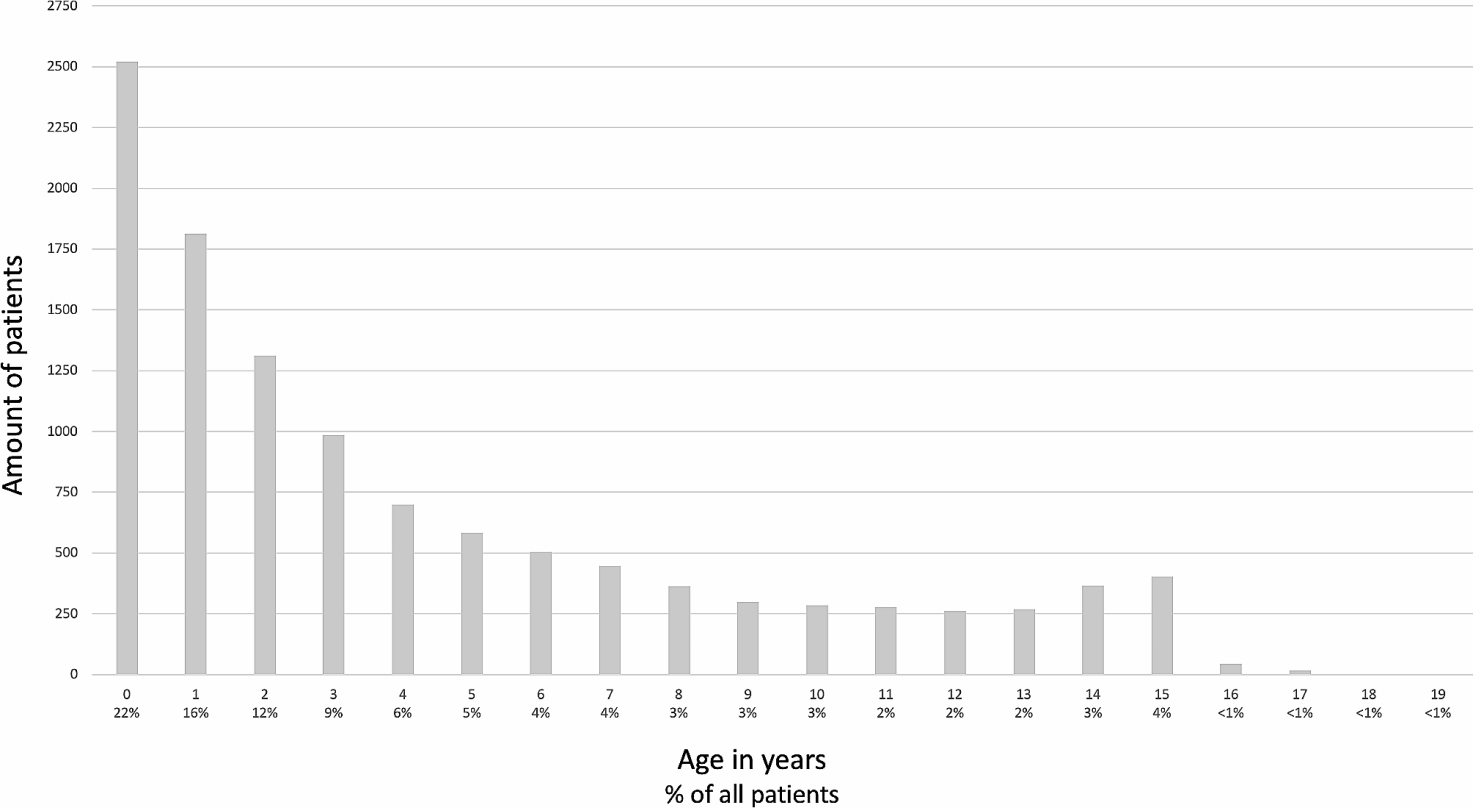
The age distribution of the 9,284 patients with 11,454 visits at the Pediatric Emergency Department of Tampere University Hospital between September 2018 and December 2019

All visits made to the Pediatric Emergency Unit of Tays Central Hospital from September 2018 to December 2019 were included in the study. The patients were 0–19.2 years of age (Fig. 1). Data were gathered retrospectively from electronic patient files in which information on all healthcare visits is routinely collected. Information collected for each visit included gender, date of visit, age at admission, level of care in the emergency department (primary vs. secondary), primary diagnosis, possible secondary diagnoses (max 2), possible hospitalization (site and length of stay), and examinations performed (laboratory, imaging, other).

For cost evaluation, the total reimbursement for the visit was calculated according to billing by the municipality. This refers to the amount that the hospital charged the municipality for a patient’s healthcare visit based on prearranged flat rates. The bottom-up costs were also collected from the hospital’s cost-per-patient software, which provided, for example, detailed costs for laboratory exams and imaging performed. Costs were estimated from the healthcare provider’s perspective. All costs are presented in 2019 euros. Costs were not adjusted for inflation due to the time limitations of the study. To equalize the purchasing power of the currency used in order to compare our results with previous research, the costs were converted to USD with a purchasing power parity (PPP) currency conversion rate from the year 2019 ($1 = €0.838) [16].

For the analyses, the data were divided into age groups of < 1-year-olds, 1–5.99-year-olds, 6–11.99-year-olds, and ≥ 12-year-olds, which are abbreviated to > 1yo, 1–5yo, 6–11yo, and > 12yo. This age-classification to infants, pre-school aged, lower elementary school aged and teenagers was conducted because these age-groups typically have similar living environments in Finalnd: infants are usually treated ate home, pre-school aged children in daycare units, and school aged at different schools depending of their age. The date of visit was used to categorize the visits by month. To compare seasonal differences, the months were divided into groups as follows. Winter: December, January, February; Spring: March, April, May; Summer: June, July, August; Autumn: September, October, November. When analyzing the diagnoses, all diagnoses with a frequency of 10 or higher were considered separately, and a category was created for diagnoses that applied to fewer than 10 cases. This category of “other” included the following diagnostic statuses: fewer than 10 cases, other suspected disease, iron deficiency anemia, atopic eczema, undefined dermatitis, urticaria, shortness of breath, undefined breathing disorder, hematuria, dizziness, fainting, feeling unwell, congenital malformation, local swelling, nephrotic syndrome, and Henoch-Shönleins purpura.

The study was carried out with the permission of the Research Director of the Hospital District of Pirkanmaa. According to Finnish law, Ethics Committee approval was not needed, as the data were drawn from existing sources and patients were not contacted.

### Statistics

The data were analyzed using IBM SPSS statistical software, version 26 (IBM Corp., New York, USA), and Microsoft Office Excel for Office 365. All variables were non-normally distributed; therefore, medians with lower and upper quartiles (Q_1_–Q_3_) were used for descriptive purposes. To compare two categorical variables, crosstab analysis and chi-square tests were used. The Mann–Whitney U test was used to compare categorical and continuous variables, and Kruskall–Wallis one-way analysis of variance was used to compare more than two groups. To determine correlations, Spearman’s rank correlation coefficient was implemented.

## Results

There were 11,454 visits and 9,284 individual patients in the study population. Over half (53.4%) of the patients were boys, and the median age was 3.01 (Q_1_- Q_3;_ 1.18–7.32) years (Table 1). More than half of the visits (52.6%) were made to secondary care treatment, and 28.1% of the patients were hospitalized (Table 1). There were significant differences between primary and secondary care treatment in terms of median length of stay (1 h 36 min [57 min–2 h 36 min] vs. 3 h [1 h 54 min–4 h 18 min], *p* <.001) and hospitalization rates (10.6% vs. 43.8%, *p* <.001) (Table 1).

**Table 1:** Characteristics of the 11,454 patient visits made to the Pediatric Emergency Department of Tampere University Hospital between September 2018 and December 2019

**Number of visits**	**n (%)**
All	11,454
Secondary Care	6,026 (52.6)
Primary Care	5,428 (47.4)
**Age of patients (yr)**	**median (Q**_**1**_ **;Q**_**3**_ **)**
All	3.1 (1.2; 7.3)
Secondary Care	3.2 (1.1; 8.5)
Primary Care	3.0 (1.3; 6.4)
**Length of visit (h)**	**median (Q**_**1**_ **;Q**_**3**_ **)**
All	2.3 (1.3; 3.6)
Secondary Care	3.0 (1.9; 4.3)
Primary Care	1.6 (1.0; 2.6)
**Gender (female)**	**n (%)**
All	5,336 (46.6)
Secondary Care	2,881 (47,8)
Primary Care	2455 (45,2)
**Number of hospitalized patients**	**n (%)**
All	3,213 (28.1)
Secondary Care	2,640 (43.8)
Primary Care	573 (10.6)

**Fig. 2:**
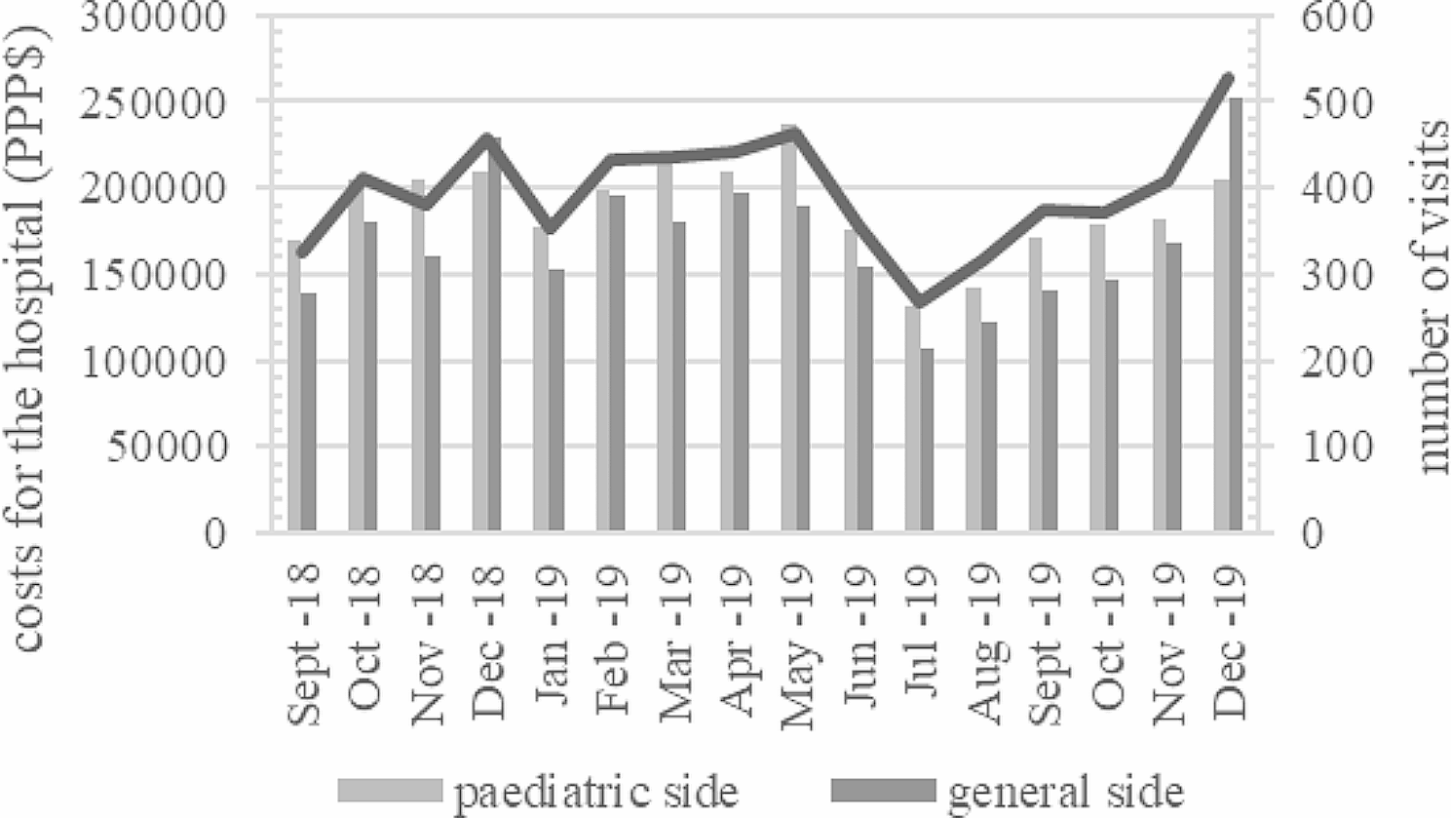
Costs for the hospital and number of visits per month in a paediatric emergency department with paediatric specialty care and primary emergency care visits. Costs are presented in US dollars with a conversion from Euros using Purchasing power parities currency conversion rate (PPP) from the year 2019

For the ED visits, the median reimbursement was €361 ($302.50), and the median bottom-up costs were €260 ($217.90). The total reimbursement amount during the study period was €3,771,691 ($3,160,677), and the costs were €3,386,346 ($2,837,758) (Table 2). Primary care had a statistically significant lower median cost than secondary care (€260 [$217.90] vs. €290 [$243.00], *p* <.001), and hospitalized patients incurred higher costs than non-hospitalized patients (€288 [$241.30] vs. €260 [$217.90], *p* <.001) (Table 2). The costs did not vary significantly between age groups (data not shown). There was no correlation between the length of visit and the cost per visit. The seasonal variations in the number of visits and the total costs are presented in Fig. 2. The largest number of patient visits was noted in December 2019 (10.8% of all visits that year) (Fig. 2).

**Tabke 2:** Costs of 11,454 paediatric emergency room visits in a finnish paediatric emergency care unit providing both primary and secondary care for children. Costs are presented in US dollars with a conversion from Euros using Purchasing power parities currency conversion rate (PPP) from the year 2019

	Median costs $ (Q_1_;Q_3_)
All visits	218 (218; 259)
Level of care	
Primary, *n*=6,026	218 (218; 224)
Secondary, *n*=5,428	243 (218; 282)
By disposition	
Discharged, *n*=8,218 ^a^ Hospitalised, *n*=3,212 ^b^	218 (218; 250) 325 (218; 279)
Total costs for the hospital	Total costs $
All visits	2,837,758
Imaging Laboratory exams	89,415 214,167
Total reimbursement	3,160,678

^a^: missing 23, ^b^: missing 1

During the study period, the most common reasons for pediatric ED visits were respiratory tract infections, counseling (the patients did not meet a doctor but received counselling from trained nurses), other infections, and gastrointestinal tract symptoms. The most common diagnoses varied between age groups, levels of care, and seasons; these are discussed in greater detail in the Supplementary Material. The prevalence of respiratory tract infections was 15.9–45.4%, depending on the age group, season, and level of care. Respiratory tract infections decreased in prevalence with age in patients over 1 year old, and the prevalence was the lowest during the summer. Counseling was a common diagnosis group, with a prevalence as high as 15.5% in primary care (see Supplementary Material). The hospitalization rate was highest during the summer and lowest during the winter (29.9% vs. 25.3%, *p* <.001), and higher during the week than on weekends (29.9% vs. 24.0%, *p* <.001). Between the diagnosis groups, hospitalization rates were highest for ataxia (100%), RSV-bronchiolitis (92.9%), diabetic ketoacidosis (87.5%), alcohol intoxication (87.0%), and diabetes (79.0%).

Around half of the patients had at least one laboratory exam conducted. The most common laboratory exams were blood count, CRP, and sodium and potassium levels; combined, they accounted for 12.0% of the costs of laboratory exams. Imaging was conducted on 13.1% of the patients. The most common type of imaging, a chest radiograph, was performed on 67.9% of the patients, although it accounted for only 38.7% of the costs of imaging (Table 3). Imaging was the most common in the 6–11yo group and least common in the < 1yo group (15.0% vs. 9.7%, *p* <.001), and more common in secondary care than in primary care (19.6% vs. 5.8%, *p* <.001). Laboratory exams were also more common in secondary than in primary care (60.0% vs. 24.1%, *p* <.001).

**Table 3:** Costs for the hospital and amounts of laboratory exams and imaging in a paediatric emergency care unit in Finland with 11,454 visits between September 2018 and December 2019

Laboratory exams					Imaging				
Visits with laboratory exams	n	% of cases			Visits with imaging	n	% of cases		
All	4,922	100			All	1,496	100		
By diagnose					By diagnose				
Other	705	14.3			Pneumoniae	221	14.8		
URTI^a^	344	7.0			Other	215	14.4		
Counselling^b^	332	6.8			Non-specific fever	120	8.0		
Non-specific fever	323	6.6			URTI	115	7.7		
Other illness	293	6.0			Counselling	87	5.8		
Pneumoniae	204	4.1			Otitis	86	5.8		
Number of laboratory exams	n	% of cases	Costs $ ^c^	% of costs	Number of imaginations	n	% of cases	Costs $	% of costs
All	14,187	288	60,017	100	All	1,689	113	94,705	100
By type					By type				
Blood count	2640	53.6	3540	5.9	Chest radiograph	1,016	67.9	36,611	38.7
CRP^c^	2322	47.2	2140	3.6	Abdominal ultrasound	315	21.1	22,437	23.7
Na, K	1837	37.3	1539	2.6	Neck ultrasound	84	5.6	5,068	5.4

^a^: upper respiratory tract infection, ^b^:The patients did not meet a doctor but received counselling from trained nurses ^c^: c-reactive protein, ^d^: Purchasing power parities currency conversion rate

## Discussion

In this retrospective register-based study, we evaluated the costs associated with and reasons for health services use in pediatric emergency care in Tampere, Finland, with two main results. First, the average cost per visit was higher for children treated in secondary care than in primary care and higher for patients requiring hospitalization than for those treated in primary care or discharged from the emergency unit. Second, infections were the most common reason for seeking medical advice, although there was variation in diagnoses by age group, sex, and season.

During the study period, there were 11,454 visits, and the total costs were over €3,380,000 ($2,837,758). The median cost per visit was €260 ($217.90) in the present study, which was more than reported in previous research. Previously, the costs associated with emergency care were evaluated in a Taiwanese descriptive study including 764,598 pediatric emergency care visits in children under 18 years in 2000–2009 [2]. It was found that the median cost per visit for all age groups was $67; for visits requiring hospitalization, it was $642, and for the non-hospitalized, $44. There were no significant differences in the costs of non-hospitalized patients of different age groups, but higher costs were associated with the infant age group among hospitalized patients [2]. A study from Portugal that included 18,111 visits compared the effects of having pediatric consultants dedicated to the emergency department versus only general pediatric consultants [17]. The findings showed that having pediatric emergency-specific consultants slightly reduced the costs per patient from €37.87 to €31.97. The difference between the costs reported in our study and other studies might be explained by differences in the billing systems in place, which make it difficult to compare the results of studies conducted in different countries. However, our results are comparable to another recently published population-based cohort study that included 4,621 ED visits (secondary/tertiary level care) in Oulu, Finland, where the mean cost per visit was €332 ($ 278.20) [18].

Our study found that a significant proportion of visits occurred due to gastrointestinal tract infections. Children’s ED visits for infections incurred a median charge of $718 ($406–$1,222 IQR) in the United States in 2011 [19]. A study conducted in the United Kingdom in 2012–2013 reported a median cost of £31–£76 per case for ED visits for fever [20].

In this study, the most common diagnoses leading to emergency care visits by children were respiratory tract infections, followed by counseling, other infections, and gastrointestinal tract symptoms. Previous studies have reported similar results. In the previously mentioned study from Finland, the most common diagnoses in the ED were respiratory tract infections, enteritis, and other viral infections [18]. In the Taiwanese study described above, the most common diagnoses for non-hospitalized patients were upper respiratory tract infection, gastrointestinal illness, and general symptoms, and regarding the hospitalized patients’ fluid and electrolyte disorders, bronchopneumonia and gastroenteritis were the most frequent [2]. In the abovementioned Portuguese study, the most common disorders were signs and symptoms (23.2%), respiratory system disorders (19.3%), and infectious disorders (16%) [17].

Some previous studies have reported different reasons for ED visits. A study from the United States including 90,236 visits to the National Hospital Ambulatory Medical Care Survey (NHAMCS) during 2001–2010 found that the most common diagnostic groups were trauma (28.2–24.9%), dental and mouth diseases (19.4–17.7%), gastrointestinal disorders (9.8%), and respiratory diseases (9.2–8.6%) [1]. A more recent study that examined NHAMCS data from 2015 found that the most common reasons for visits were respiratory disorders, injury and poisoning, nervous system disorders, and digestive disorders [12]. A large study in Italy, covering 1.6 million patients under 18 years old, found the most common diagnoses to be injuries (26%), respiratory tract infections (22%), and gastrointestinal disorders (8%) [21]. These findings show that there is variability in the reasons for pediatric emergency care visits, but also that the number of visits for respiratory tract infections remains high in a variety of settings. The number of injuries, incidents of trauma, or dental emergencies is not comparable to the present study population because most of these cases are not treated in the pediatric emergency unit in our hospital.

Our study showed that the highest costs and the most visits occurred in December, which is one of the winter months in Finland. This finding is intuitive, as respiratory infections were one of the most common reasons for emergency care visits, and respiratory tract infections are most prevalent during winter. In a study conducted in the United States, winter was the peak season for pneumonia, otitis media, and upper respiratory tract infections [22]. Also in line with our findings, a study performed in the United States in 2011 estimated that 28% of all pediatric ED visits were made because of infections and that more than double the number of infections were treated in the ED in February compared to July [19]. Moreover, similar findings were noted in other previously published studies [2, 12, 21]. 

The admissions rate of our study was 28.1%, whereas in other studies on pediatric emergency care, the rate varied from 6–24% [20, 23, 24, 25]. The rate of admissions could be seen as a marker for determining how emergent the visits were. This would then indicate that the system that directs patients to the Pediatric Emergency Unit of Tays Central Hospital is appropriate and, moreover, ensures that the number of patients who truly require emergent care, as a percentage of admissions, is similar or higher than in other pediatric emergency departments. This is further supported by the finding that the admissions rate in this study was higher on weekdays than weekends as the path of care was different, as explained in the methods.

The main strength of this study is the quality of the study material. In Finland, extensive information is routinely gathered on all patients; therefore, all visits made to the emergency department could be included in the study. However, as this study is a retrospective register study, not all relevant information was available from the patients, and it is possible that there might be some errors regarding the details of the care of individual patients. Healthcare in Finland is mostly funded by taxes, and the out-of-pocket costs for patients are very low. Moreover, reimbursement paid by both the patients and the municipalities is based on fixed prices and not on the actual costs of care. This leads to small differences in the costs of visits and decreases the generalizability of the results to different healthcare systems in different countries. The reasons behind visits and other results not regarding costs, however, can be generalized to represent the pediatric ED population more widely.

In conclusion, we found only small differences in the costs of emergency care visits, but higher costs were related to more seriously ill children treated in secondary care in the emergency department or admitted to inpatient treatment. The number of patients followed a seasonal variation, mainly due to the epidemiologic variance in respiratory tract infections.

### Electronic supplementary material

Below is the link to the electronic supplementary material.

Supplementary Material 1

## Data Availability

The datasets used and/or analysed during the current study are available from the corresponding author on reasonable request.
