# Supplementary material for: An analysis of the diagnoses and costs of pediatric emergency care visits: a single center study
Source: BMC Health Serv Res. 2024 Feb 27;24:251. doi: 10.1186/s12913-024-10746-1 (PMC10900614; doi:10.1186/s12913-024-10746-1)
Supplement: Supplementary file 1 — Supplementary Material 1 [file 12913_2024_10746_MOESM1_ESM.docx]

| Side of care | n | % of cases | Age groups | n | % of cases | Seasons* | n | % of cases |
| --- | --- | --- | --- | --- | --- | --- | --- | --- |
| All visits | 11,454 | 100 | <1-year-olds | 2,320 | 100 | Winter | 2,325 | 100 |
| Respiratory tract infection | 4,186 | 36.5 | Respiratory tract infection | 947 | 37.6 | Respiratory tract infection | 933 | 40.1 |
| Other | 2,519 | 22 | other | 627 | 24.9 | Other | 540 | 23.2 |
| Counselling | 1,133 | 9.9 | Counselling | 328 | 13.0 | Counselling | 178 | 7.7 |
| Other infection | 983 | 8.6 | Non-specific symptom during infancy | 188 | 7.5 | Other infection | 172 | 7.4 |
| GI-tract symptom | 444 | 3.9 | Other infection | 140 | 5.6 | Gastroenteritis | 108 | 4.6 |
| General side | 5,428 | 100 | 1-5-year-olds | 5,393 | 100 | Spring | 2,458 | 100 |
| Respiratory tract infection | 2,399 | 44.2 | Respiratory Tract infection | 2,451 | 45.4 | Respiratory tract infection | 863 | 35.1 |
| Other | 1,067 | 19.7 | Other | 837 | 15.5 | Other | 464 | 18.9 |
| Counselling | 841 | 15.5 | Counselling | 549 | 10.2 | Counselling | 376 | 15.3 |
| Other infection | 446 | 8.2 | Other infection | 539 | 10.0 | Other infection | 184 | 7.5 |
| Gastroenteritis | 179 | 3.3 | Gastroenteritis | 262 | 4.9 | Gastroenteritis | 117 | 4.8 |
| Paediatric side | 6,026 | 100 | 6-11-year-olds | 2,179 | 100 | Summer | 1,666 | 100 |
| Respiratory tract infection | 1,787 | 29.7 | Other | 611 | 28.0 | Other | 469 | 28.2 |
| Other | 1,452 | 24.1 | Respiratory tract infection | 571 | 26.2 | Respiratory tract infection | 407 | 24.4 |
| Other infection | 537 | 8.9 | Other infection | 207 | 9.5 | Other infection | 203 | 12.2 |
| Neurological disease | 318 | 5.3 | Counselling | 198 | 9.1 | Counselling | 203 | 12.2 |
| GI- tract symptom | 301 | 5.0 | Gi- tract symptom | 141 | 6.5 | GI- tract symptom | 77 | 4.6 |
|  |  |  | >12-year-olds | 1,362 | 100 | Autumn | 1,975 | 100 |
|  |  |  | Other | 444 | 32.6 | Respiratory tract infection | 658 | 33.3 |
|  |  |  | Respiratory tract infection | 217 | 15.9 | Other | 402 | 20.4 |
|  |  |  | Poisoning | 144 | 10.6 | Counselling | 239 | 12.1 |
|  |  |  | Other infection | 97 | 7.1 | Other infection | 167 | 8.5 |
|  |  |  | Psychiatric disorder | 78 | 5.7 | GI- tract symptom | 98 | 5.0 |
|  |  |  |  |  |  |  |  |  |

Supplementary material 1. Most common diagnoses in different groups of patients in paediatric emergency care unit in Tampere Finland. The unit functions as a primary emergency care unit and a secondary emergency care unit for patients needing paediatric specialty care.

When analysing the diagnoses, all diagnoses with a frequency of 10 or more were taken into consideration separately and a category for the diagnoses that had less than 10 cases was created. The category of ‘’other’’ includes diagnoses: less than 10 cases, other suspected disease, iron deficiency anaemia, atopic eczema, undefined dermatitis, urticaria, shortness of breath, undefined breathing disorder, haematuria, dizziness, fainting, feeling unwell, congenital malformation, local swelling, nephrotic syndrome and Henoch-Shönleins purpura.
